# Marine Renewable Energies: Perspectives and Implications for Marine Ecosystems

**DOI:** 10.1155/2013/547563

**Published:** 2013-06-19

**Authors:** Arianna Azzellino, Daniel Conley, Diego Vicinanza, Jens Peter Kofoed

**Affiliations:** ^1^Environmental Engineering Division, DICA-Civil and Environmental Engineering Department, Politecnico di Milano, 20133 Milano, Italy; ^2^Department of Civil Engineering, Aalborg University, 9000 Aalborg, Denmark; ^3^School of Marine Science and Engineering, Plymouth University, Plymouth PL4 8AA, Devon, UK; ^4^Department of Civil Engineering, SUN, Seconda Università di Napoli, 81031 Aversa, Caserta, Italy

Countries with coastlines may have valuable renewable energy resources in the form of tides, currents, waves, and offshore wind. The potential to gather energy from the sea has recently gained interest in several nations [1–3], so Marine Renewable Energy Installations (hereinafter MREIs) will likely become very diffuse in the near future and determine a further transformation of our coastal seas. 

Coastal zones are, in fact, already subjected to significant pressure from human activities, as a result of their high biological productivity and accessibility. It might be expected that the MRE sector development will add its impacts to those of the existing pressures. 

Up to now the public concern about the environmental impacts of renewable energy projects has been a major factor behind the stalling or rejection of many planning applications for on-shore renewables developments. Siting renewables facilities in off-shore locations would appear to reduce this tension [4], but it cannot be forgotten that coastal ecosystems have already experienced major changes due to human activities, while the spatial conflicts of sea uses and demands are increasingly growing. In such a complex framework of existing uses, pressures, and foreseen developments, the MRE sector development makes urgent the use of Marine Spatial Planning approaches. Spatial decision support systems, through the efficient exchange of information between experts, stakeholders, and decision makers, offer the opportunity to guide the transition from the single sector management toward the integrated management of sea uses.

Concerning the marine realm, in fact, the integration of the resource planning has become a sought-after norm after the many failures of the traditional sectoral, single-issue management. Fisheries collapse, threats to marine biodiversity, and global climate change effects are all elements that require a greater integration in marine resource management and policies. Moreover, the greater awareness of the extent to which our marine habitats have become degraded, the widening of interests in—and users of—the marine space, including the general public, and the increased governmental commitment to a wider stakeholder participation in marine decision-making have created the ground for marine spatial planning becoming essential for analysing and allocating the spatial and temporal distribution of human activities in marine areas, in order to comply with fixed ecological, economic, and social objectives.

In this framework, the knowledge on the potential environmental risks that might be associated with the presence of MREIs, the prediction of the areas of particularly vulnerable environmental characteristics, and the early identification of conflictual uses will feed the spatial planning process and create the ground for mitigation actions or early negotiations between stakeholders.

To date only few studies have considered the potential environmental risks associated with the presence of MREIs. 

The fact that many MRE devices are still in the experimental/trial phase is the reason why no data are available on the environmental effects of commercial developments and why presently it is not fully clear how to scale up from the limited observations on individual or small clusters of devices to commercial scale arrays. 

The offshore wind industry, now extensive and well established, has already taught numerous lessons regarding monitoring methodologies and key receptors; however, to establish the baseline conditions of a site in order to evaluate impacts remains the critical point.

The articles contained in this special issue build further on the idea of the knowledge basis needed to accelerate the implementation of spatial planning decision support tools in the context of the management and, based on their particular field of expertise, provide a perspective on needs and opportunities offered by the MRE sector development. 

The contributions consider various elements of the environmental impact assessment, spanning from the assessment of baseline conditions, the identification of control sites, the design of monitoring protocols, the need to combine the information derived by different MRE projects, and the perceived necessity to move towards adaptive management schemes that may benefit from the progress in the knowledge acquisition.

Effective and reliable decision-making needs sound research. In their article: “*Epibenthic assessment of a renewable tidal energy site*,” E. V. Sheehan et al. provide a baseline benthic survey for the Big Russel in Guernsey, UK, a potential site for tidal energy development. They compared the abundance of organisms on different habitat types and the assemblage composition of sites within the Big Russel in order to assess the suitability of a previously suggested control site and other potential locations for devices. Their baseline survey is meant to be used to select control habitats with which to compare and monitor the benthic communities after installation of devices and contribute towards the optimal siting of any future installation.

A common feature of environmental impact assessment studies is the need to compare alternative scenarios, and this may be done by using a simulation approach or using the information derived from different MRE projects.

In their paper “*The environmental impact of a Wave Dragon array operating in the Black sea*,” S. Diaconu and E. Rusu discuss the influence on the shoreline dynamics of a potential Wave Dragon installation in the Black sea. They use a simulation approach and evaluate the impact of the wave energy farm in the two representative scenarios: (1) scenario without any wave energy converter and (2) scenario of a Wave Dragon installation consisting of six wave energy converters. Their results show that the presence of the MREI has a significant influence near the wave farm that gradually decreases towards the coastline. They also analyse the influence of the WEC array on longshore currents, using a nearshore circulation model and found the longshore current velocities to be more affected by the presence of the wave farm than the significant wave height. The authors discuss also how effects may possibly impact the marine flora and fauna.

In their paper: “*Differentiating between underwater construction noise of monopile and jacket foundations for offshore windmills: A case study from the Belgian part of the North Sea,*” A. M. J. Norro et al. compare the underwater noise generated during the piling activities of steel monopiles at the Belwindwind farm (Blighbank) with that of jacket pinpiles at the C-Power project (Thorntonbank). Underwater noise is measured at various distances from the pile driving location. In their study, no significant differences are found between monopile and jacket pinpiles, having nearly identical spectra. The implications for the windmills construction are not insignificant, being the piling of the jacket pinpiles 2.5 timefolds more time consuming than monopile and requiring more energy. The implications of the underwater noise production are also evaluated in terms of radius of major behavioural disturbance for the sensitive species, the harbour porpoise, *Phocoena phocoena, *being found as almost the same for the two types of piling.

MREI may also produce positive impacts in the marine ecosystem, acting as artificial reefs, and offer the opportunity of strengthening MRE planning applications by combining energy production with other marine productions. In her review article “*Artificial reef effect in relation to offshore renewable energy conversion*,” Langhamer discusses the opportunities offered by MREIs in terms of habitat enhancement for threatened or commercial interesting species. She describes why it is highly possible that offshore energy installations act as artificial reefs and may support both environmental and commercial interests. However, she points out that the lack of basic knowledge is very often the reason why artificial reefs may fail to enhance biomass production. Detailed ecological studies testing the enhancement potential of different types and dimensions of scour protection would be necessary, before developing management criteria (i.e., no-take zones for fisheries). Besides illustrating the economic opportunities of combining different farming systems (e.g., mussel farming and seaweed cultivation) with the existing offshore parks, Langhamer discusses how further research work may strengthen planning applications for future developments, based also on the cooperation of different MREIs, collecting environmental data using a Before-and-After-Control-Impact design, option that may significantly accelerate application processes and reduce the need to repeat studies.

Adaptive management is becoming a diffuse framework of choice for environmental management. Whether active (i.e., based on deliberate experimentation with alternative environmental management approaches whose impact is evaluated) or passive (based on a single management approach for which the impact is predicted and then monitored), the updating of the conceptual understanding of the impacts and the response of the natural systems to management interventions offer the opportunity to shape the management schemes (and in the monitoring itself) to what is suggested by evidences brought by the initial monitoring. In their paper “*An adaptive framework for selecting environmental monitoring protocols to support ocean renewable energy development*,” E. J. Shumchenia et al. discuss an adaptive framework based on indicators of the likely changes to the marine ecosystems due to MREIs and develop decision trees to identify impacts, at both the demonstration and commercial scales, as function of type of energy (e.g. wind, tidal, or wave), structure (e.g., turbine), and foundation type (e.g., monopole). In their study, impacts are categorized by ecosystem component (i.e., benthic species, fish, birds, marine mammals, and sea turtles) and monitoring objectives are developed for each. In consideration of the poor knowledge about the baseline natural variability of the environmental indicators and the difficulties of separating impacts from the noise of the seasonal or interannual environmental variability, these authors propose an adaptive monitoring framework, as alternative to the more diffuse “static” type, since it might benefit from the progress in the knowledge acquisition and improved understanding of the impacts on marine resources deriving from the initial monitoring activity, which may, on its turn, greatly change the case specific monitoring needs and/or requirements.

All the papers in this issue are intended to advance more strategic and integrative thinking on how to apply an ecosystem-based spatial planning approach to better manage the integration of the MRE sector development into the existing framework of human sea uses. The growing concern over the threat of global climate change and the other environmental impacts of the worldwide reliance on fossil fuels have amplified the interest on renewable energies and drawn the attention on the immense stores of energy in the ocean [5]. Advocates of renewable energies endorse their multitude of economic and energy security benefits compared to other sources of conventional electricity generation and ground their reasons on the global benefit of reducing carbon emissions and on the collateral benefits of the lower consumption and pollution of water resources.

Notwithstanding, environmentalists and some environmental scientists have criticized the very diffuse wind energy installations, both terrestrial and marine, for their negative impacts on wildlife, and especially birds. In this respect we believe that the environmental concerns should not hinder the future of the MRE sector in absolute terms but instead foster the developing of guidelines to properly conduct the environmental impact studies, aiming to maximise the protection of the marine environment.

Sovacool [6] in a recent paper argues that conventional electricity systems, as nuclear power and fossil-fuelled power systems, have also a host of environmental and wildlife costs, particularly for birds. Through a coarse calculation of the avian fatalities of wind electricity, fossil-fueled, and nuclear power systems across the entire United States, Sovacool estimated that the risks to wildlife and birds, due to conventional electricity systems, are far greater than those from wind energy. His analysis reminds us that when dealing with environmental impact assessment issues that “by definition” need to be conducted using a relative scale of reference, we need always to consider the whole picture.

So if we are evaluating the avian fatalities due to wind energy installations, we cannot forget the higher number of avian deaths that may be accounted to fossil fuels as result of climate change global effects, or the other collateral impacts causing habitat alterations or contamination of land and water. We should not expect that low-emission, low-pollution energy sources will have no environmental impacts, but we have to assess instead that their impacts will be lower than the one of the conventional sources and acceptable in the perspective of the ecological sustainability.

*Arianna Azzellino* *Arianna Azzellino* *Daniel Conley* *Daniel Conley* *Diego Vicinanza* *Diego Vicinanza* *Jens Peter Kofoed* *Jens Peter Kofoed*
